# Social isolation and physical distance: Experiences from a phone pal

**DOI:** 10.1192/j.eurpsy.2021.208

**Published:** 2021-08-13

**Authors:** M. Pinto Da Costa

**Affiliations:** Unit For Social And Community Psychiatry (who Collaborating Centre For Mental Health Services Development), Queen Mary University of London, London, United Kingdom

## Abstract

**Abstract Body:**

People with psychosis are commonly socially isolated, both due to their condition, and the stigma towards them. Remote volunteering over smart-phone can be a way to overcome social isolation and physical distance, promoting social inclusion. This talk will present the qualitative findings from a feasibility study – the Phone Pal – which connected in the United Kingdom patients with psychosis with community volunteers, to communicate with each other for up to 12 weeks via smart-phone (through texts, WhatsApp messages, e-mails, audio or video calls). Participants described at the end of the study their experiences of communicating with their match over the smart-phone in terms of frequency, duration and timing of communication, their communication method, content and style, and the changes of communication over time. Several participants reported a positive impact of being connected with someone, meeting a new person, feeling supported and feeling better, and a few described challenges, such as disappointment, guilt and burden. These interview findings show that some matches were able to develop a positive and friendly relationship, and were willing to continue to be in contact with each other beyond the study duration. It is hoped that this talk will generate a lively discussion, gathering further understanding about the potential benefits and challenges of remote volunteering over smart-phone for patients and volunteers, and its potential usefulness in the current pandemic times.

**Disclosure:**

No significant relationships.

